# Identification of a pluripotency-inducing small compound, PLU, that induces callus formation *via* Heat Shock Protein 90-mediated activation of auxin signaling

**DOI:** 10.3389/fpls.2023.1099587

**Published:** 2023-03-08

**Authors:** Yuki Nakashima, Yuka Kobayashi, Mizuki Murao, Rika Kato, Hitoshi Endo, Asuka Higo, Rie Iwasaki, Mikiko Kojima, Yumiko Takebayashi, Ayato Sato, Mika Nomoto, Hitoshi Sakakibara, Yasuomi Tada, Kenichiro Itami, Seisuke Kimura, Shinya Hagihara, Keiko U. Torii, Naoyuki Uchida

**Affiliations:** ^1^ Center for Gene Research, Nagoya University, Nagoya, Japan; ^2^ Graduate School of Science, Nagoya University, Nagoya, Japan; ^3^ School of Science, Nagoya University, Nagoya, Japan; ^4^ Center for Sustainable Resource Science, RIKEN, Saitama, Japan; ^5^ Institute of Transformative Bio-Molecules, Nagoya University, Nagoya, Japan; ^6^ Institute for Advanced Research, Nagoya University, Nagoya, Japan; ^7^ Center for Sustainable Resource Science, RIKEN, Yokohama, Japan; ^8^ Graduate School of Bioagricultural Sciences, Nagoya University, Nagoya, Japan; ^9^ Department of Industrial Life Sciences, Faculty of Life Science, Kyoto Sangyo University, Kyoto, Japan; ^10^ Center for Plant Sciences, Kyoto Sangyo University, Kyoto, Japan; ^11^ Howard Hughes Medical Institute and Department of Molecular Biosciences, University of Texas at Austin, Austin, TX, United States

**Keywords:** *Arabidopsis thaliana*, auxin, callus, HSP90, pluripotency, small compound

## Abstract

Plants retain the ability to generate a pluripotent tissue called callus by dedifferentiating somatic cells. A pluripotent callus can also be artificially induced by culturing explants with hormone mixtures of auxin and cytokinin, and an entire body can then be regenerated from the callus. Here we identified a pluripotency-inducing small compound, PLU, that induces the formation of callus with tissue regeneration potency without the external application of either auxin or cytokinin. The PLU-induced callus expressed several marker genes related to pluripotency acquisition *via* lateral root initiation processes. PLU-induced callus formation required activation of the auxin signaling pathway though the amount of active auxin was reduced by PLU treatment. RNA-seq analysis and subsequent experiments revealed that Heat Shock Protein 90 (HSP90) mediates a significant part of the PLU-initiated early events. We also showed that HSP90-dependent induction of *TRANSPORT INHIBITOR RESPONSE 1*, an auxin receptor gene, is required for the callus formation by PLU. Collectively, this study provides a new tool for manipulating and investigating the induction of plant pluripotency from a different angle from the conventional method with the external application of hormone mixtures.

## Introduction

Since plants are sessile organisms, they have developed mechanisms thorough evolution that enable flexible responses to ever-changing internal and external situations. One of such abilities is a high degree of plasticity for changing developmental programs (Gaillochet and Lohmann, 2015). Plants can generate a pluripotent tissue called callus by dedifferentiating somatic cells (Ikeuchi et al., 2016; Perez-Garcia and Moreno-Risueno, 2018; Eshed Williams, 2021). In nature, a callus is a mass of highly proliferative cells mainly for injury repair and occasionally acts as a site for pluripotency acquisition to regenerate organs. A pluripotent callus can also be artificially induced from explants using a callus-inducing medium (CIM) which contains appropriate concentrations of auxin and cytokinin at a high auxin/cytokinin ratio (Skoog and Miller, 1957; Ikeuchi et al., 2013; Shin and Seo, 2018). Shoot tissues can then be regenerated by transferring the callus to a cytokinin-rich shoot-inducing medium (SIM). This methodology has been widely used, from basic genetic technologies to agricultural applications. However, because the success of callus induction for many plant species still requires time-consuming attempts with changing the amount and ratio of applied hormones for each species, the development of alternative pluripotency induction methodologies will help overcome this situation by complementing the conventional methodology.

Callus induction by CIM partly shares processes with lateral root formation (Atta et al., 2009; Sugimoto et al., 2010). The formation of lateral root primordia is initiated by the division of differentiated pericycle cells adjacent to protoxylem cells, called xylem pole pericycle (XPP) cells (Peret et al., 2009). CIM induces the formation of XPP-like cells even if the starting explant is derived from any organs, and the formed callus initial exhibits a lateral-root-primordia-like structure (Atta et al., 2009; Sugimoto et al., 2010). CIM-induced callus formation is suppressed in mutants incapable of lateral root initiation, indicating that CIM requires the activation of a lateral root development program for callus induction. The ability of auxin to trigger lateral root initiation (Fukaki et al., 2005; Okushima et al., 2007; Goh et al., 2012) underlies the fact that auxin is an essential ingredient of CIM to initiate callus formation. Callus tissues express genes involved in the formation and maintenance of root meristem such as *WUSCHEL-RELATED HOMEOBOX 5* (*WOX5*) (Haecker et al., 2004), *SCARECROW* (*SCR*) (Di Laurenzio et al., 1996), *PLETHORA* (*PLT*) family (Aida et al., 2004), and these genes play important roles in establishing pluripotency (Kareem et al., 2015; Kim et al., 2018; Ikeuchi et al., 2019; Shin et al., 2020). When a callus is transferred to SIM, the established pluripotency in the callus enables shoot regeneration that reconstructs shoot meristems and develops shoot tissues. However, although molecular mechanisms for pluripotency induction have been gradually revealed, the current understanding is mainly based on reports on CIM- or wound-induced calli (Ikeuchi et al., 2016; Perez-Garcia and Moreno-Risueno, 2018; Eshed Williams, 2021). Therefore, approaches without hormone treatment or wounding may reveal as-yet-unknown mechanisms of pluripotency induction.

In this study, we identified a novel pluripotency-inducing small compound, PLU, that induces the formation of callus with tissue regeneration potency without external application of either auxin or cytokinin. PLU-induced callus formation required activation of the auxin signaling pathway despite the reduction in the amount of active auxin. Further analyses revealed that Heat Shock Protein 90 (HSP90) mediates a significant part of PLU-initiated early responses, and that PLU potentiates auxin signaling *via* activation of expression of auxin receptor genes in an HSP90-dependent manner. Collectively, this study provides novel insights and a new tool for understanding and manipulating the induction of plant pluripotency.

## Materials and methods

### Plant materials

The *Arabidopsis thaliana* Columbia (Col-0) accession was used as the wild-type background in this study, except for *J0121* in the C24 accession. *slr/iaa14* mutant, *tir1/afb*-family mutants, and marker lines of *AtHB8pro:4xYFP, SCRpro:4xYFP, SUC2pro:4xYFP, J0121* (*J0121:GFPer*), and *DR5:GUS* were described previously (Ulmasov et al., 1997; Dharmasiri et al., 2005b; Fukaki et al., 2005; Laplaze et al., 2005; Marques-Bueno et al., 2016; Prigge et al., 2020).

### Plasmid construction and generation of transgenic plants

Plasmids constructed in this study and primers used for the construction are listed in **Tables S1** and **S2**, respectively. *PLT2pro:Eluc-3xVenus*, *PLT3pro:Eluc-3xVenus*, *CUC2pro:Eluc-3xVenus* were introduced into Col, and *TIR1pro:TIR1-Venus* was introduced into *tir1 afb2*. More than fifteen T1 plants were generated for each construct, and at least two lines harboring the corresponding transgene at a single locus were selected for further analyses.

### Plant culture and compound treatment

Seeds were sterilized, stored in the dark at 4°C for a few days, transferred to a half Murashige and Skoog (1/2 MS) medium containing 0.5% sucrose, and grown under continuous white light at 22°C. For liquid culture, seeds were germinated in 24-well plates on a shaker set at 140 rpm. For chemical treatment, seedlings at 1 or 5 days post-inoculation (dpi) were treated with compounds, and samples were observed after incubation for indicated days.

### Callus formation

Seedlings were grown in solid 1/2 MS medium for 5 days, and cut at seven mm from the root tip. Root explants were transferred to CIM (1/2 MS medium containing 10 g/l sucrose, 0.5 mg/l 2,4-D and 0.1 mg/l kinetin) or 1/2 MS medium containing 10 g/l sucrose and 40 μM PLU (CAS No.1060777-62-9; Z154270794, Enamine), and cultured for 6 days. For experiments in **Figure 2A**, 20 g/l glucose was used instead of sucrose as a sugar source. The explants were then transferred to SIM (Gamborg B5 medium containing 20 g/l glucose, 0.2 mg/l indole-3-acetic acid (IAA) and 2.5 mg/l 2-isopentenyladenine.

### GUS staining

GUS staining was performed as described previously (Uchida et al., 2007). Briefly, seedlings at 5 dpi were treated with ice-cold 90% acetone for 20 min, washed with water, placed in GUS staining solution (50 mM sodium phosphate [pH 7.0], 10 mM potassium ferricyanide, and 10 mM potassium ferrocyanide, 2 mM X-gluc, 0.2% Triton-X) and vacuumed for 5 min. After incubation at 37°C, root samples were mounted in water and observed.

### Microscopy observation

For observation of cleared samples, FAA-fixed samples were washed with water and cleared with chloral hydrate solution (8 g chloral hydrate, 1ml glycerol, and 2ml water) for at least 1 day. The cleared samples were observed using an Axio Imager A2 microscope (Zeiss) and photographed with an Axiocam 512 color camera (Zeiss). Fluorescence observation was performed using the ClearSee method (Kurihara et al., 2015) with a confocal laser microscope LSM800 or LSM900 (Zeiss). YFP and GFP were excited at 488 nm, and fluorescent images were acquired with the detection range from 490 to 546 nm. Differential interference contrast (DIC) images were taken by a T-PMT detector. Image adjustments were performed using the ImageJ software.

### Yeast two hybrid assay

Yeast two hybrid assay was performed as described previously (Uchida et al., 2018). Briefly, EGY48 strain transformed with pSH18-34 (LexA operon::LacZ reporter), pGLex313/TIR1 (LexA-DNA-binding domain fused with TIR1), and pJG4-5/IAA3-DI/DII (B42-transcriptional-activator domain fused with DI/DII domain of IAA3) was incubated in liquid medium composed of minimal SD/Gal/Raf base (Clontech), –His/–Trp/–Ura dropout supplement (Clontech), 50 mM Na-phosphate buffer (pH 7.0), 80 μg/ml X-gal (Wako) and various concentrations of compounds. After 3-day incubation, cultured media containing yeast were transferred to wells in a 96-well plate (white, flat bottom) and observed.

### Quantification of endogenous auxin

Seedlings at 5 dpi were treated with 40 μM PLU and used for the quantification of IAA and its aspartic-acid-conjugated inactivated form (IAAsp). Each sample was prepared from a pool of 60 whole seedlings. Quantification was performed with ultra-high performance-liquid chromatography (UHPLC)-electrospray interface quadrupole-orbitrap mass spectrometer (UHPLC/Q-Exactive; Thermo Scientific) and an ODS column (ACQUITY UPLC HSS T3, 1.8 µm, 2.1 x 100 mm; Waters) as described previously (Kojima et al., 2009; Shinozaki et al., 2015).

### RNA-seq analysis

Seedlings at 5 dpi cultured in liquid media were treated with 40 μM PLU for one hour, and total RNAs were extracted and purified using RNeasy Plant Mini Kit (QIAGEN). Each RNA sample was prepared from a pool of 20 whole seedlings. Three independent RNA samples were prepared. 3 μg of purified RNA was used for RNA-seq as described previously (Uchida et al., 2018). The count data were subjected to the trimmed mean of M-value (TMM) normalization, and then transcript expression and digital gene expression were defined using edgeR (Robinson et al., 2010). Gene Ontology (GO) analysis was performed using the PANTHER classification system (http://pantherdb.org/). Venn diagram was drawn using the “Draw Venn Diagram” software at the Van de Peer Lab website (http://bioinformatics.psb.ugent.be/webtools/Venn/). For drawing a Venn diagram with PLU-upregulated genes, we used the publicly available reported lists of auxin-induced genes, CIM-induced genes, and Lateral Root Initiation (LRI)-related genes (Vanneste et al., 2005; Sugimoto et al., 2010; Uchida et al., 2018).

### qRT-PCR

Each total RNA was extracted from a pool of 20 whole seedlings using an RNeasy Plant Mini Kit (QIAGEN), and quantitative real-time PCR (qRT-PCR) was performed using a ReverTra Ace qPCR RT Master Mix with gDNA Remover kit (TOYOBO), a SYBR Fast qPCR kit (KAPA), and a Light Cycler 96 (Roche). The primers used for expression analyses are listed in **Table S2**.

### Cis-element analysis

292 genes significantly upregulated more than two-fold upon the PLU treatment (FDR<0.01, logFC>1) were analyzed for enriched motifs by a program that identifies overrepresented cis elements in 1-kb upstream sequences from the transcriptional start site of input genes by comparing surrounding sequences for every pentamer in the upstream sequences as previously reported (Yamamoto et al., 2011). The enriched octamers were aligned according to conserved pentamer sequences and then analyzed using Weblogo version 2.8.2 (Crooks et al., 2004).

## Results

### The small compound PLU induces abnormal venation patterns and callus formation

**Figure 1:**
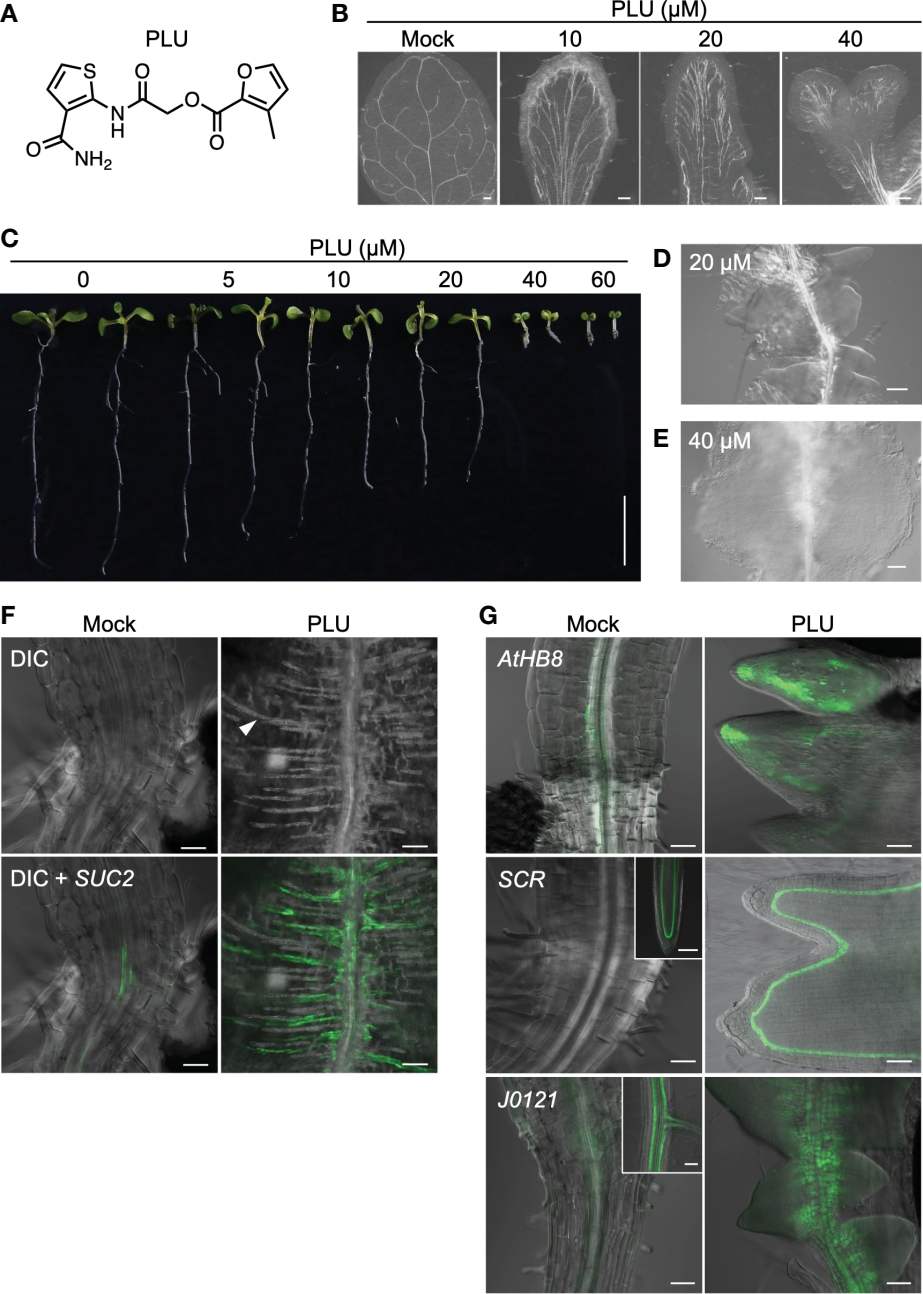
PLU induces abnormal venation patterns and the formation of callus. **(A)** The molecular structure of PLU. **(B)** Differential Interference Contrast (DIC) images of cleared true leaves of wild-type seedlings treated with DMSO (mock) or PLU from 1 to 11 days post inoculation (dpi). Scale bars: 200 μm. **(C)** PLU-treated seedlings. Scale bar: 1 cm. **(D, E)** Close-up image of roots below the hypocotyl-root junction at 20 µM treatment **(D)** and the hypocotyl-root junction at 40 µM treatment **(E)**. Scale bars: 100 μm. **(F, G)** *SUC2pro:4xYFP* reporter plants were treated with DMSO (mock) or 40 µM PLU at 1 dpi, and regions around the hypocotyl-root junction were observed at 8 dpi. Fluorescent signals of reporter lines were merged with DIC images. In DIC microscopy, thick secondary cell walls of xylem tracheary elements can be easily recognized (the white arrowhead indicates a representative signal). 20 µM PLU-treated *AtHB8pro:4xYFP*, *SCRpro:4xYFP*, and *J0121:GFPer* **(G)**. Insets show original expression patterns of the reporters in the roots of mock-treated plants. Scale bar: 50 µm.

Previously we performed a chemical screen to identify small compounds that increase the number of stomata of *Arabidopsis thaliana* seedlings using a collection of small synthetic compounds (Ziadi et al., 2017). During the screen of 11,000 compounds, we unexpectedly found that a compound composed of two heteroaromatics, furan and thiophene, tethered by a spacer (formally, 2-Furancarboxylic acid, 3-methyl-, 2-[[3-(aminocarbonyl)-2-thienyl]amino]-2-oxoethyl ester) (**Figure 1A**), affects leaf venation patterns. We named this compound PLU (Pluripotency-inducing compound; see the later explanation). When wild-type seedlings were incubated with different concentrations of PLU in liquid culture, true leaves exhibited abnormal venation patterns in a dose-dependent manner (**Figure 1B**). Upon 10 µM PLU treatment, vascular strands were formed parallel in the central regions, and leaves were occasionally fused when treated at concentrations higher than 20 µM. Cotyledons of PLU-treated plants ectopically developed many short secondary free-ending veins from primary loop-shaped veins (**Figure S1**).

PLU treatment also influenced the growth of the whole seedling body (**Figure 1C**). Root growth was attenuated in a dose-dependent manner, and growth of both aboveground tissues and roots was severely inhibited at higher than 40 µM. Intriguingly, 20 µM treatment led to the formation of structures that appeared to consist of closely formed multiple lateral root primordia along the primary root just below the hypocotyl-root junction (**Figure 1D**), and 40 µM treatment induced a large cell mass, or callus (**Figure 1E**).

Close observation of the PLU-induced callus showed the ectopic formation of xylem strands, which ran in the direction vertical to the original vascular bundle of the primary root (**Figure 1F**). *SUC2pro:4xYFP*, a marker of mature phloem companion cells, was also expressed along the ectopically formed xylem strands in the PLU-induced callus. *AtHB8* (*AtHB8pro:4xYFP*), a marker gene for vascular stem cells (procambial cells) that produce both xylem and phloem (Baima et al., 1995) was expressed around the central part of root vasculatures in mock-treated plants, while it was broadly expressed in the PLU-induced callus (**Figure 1G**). In this callus, *SCR*, which is required for the proper maintenance of root meristem (Di Laurenzio et al., 1996), and *J0121*, an enhancer-trap line for XPP cells acting as initial cells for lateral root formation (Laplaze et al., 2005), were also expressed (**Figure 1G**). *SCR* (*SCRpro:4xYFP*) was expressed in quiescent center cells and developing endodermal cells at the root tip in mock-treated plants, and the expression disappeared in mature regions, including the hypocotyl-root junction, while it was expressed in the PLU-induced callus, at one layer located a-few-cell apart from the epidermis. *J0121* (*J0121:GFPer*) was observed as two strands of XPP cells along vasculatures in developing roots of mock-treated plants, and the signal gradually became dim toward the hypocotyl. In the PLU-induced callus, the *J0121* signals were clearly detected and expanded toward the cell mas beyond the original expression domain. These results suggested that the PLU-induced callus may retain some characteristics related to root meristem and lateral root development.

### PLU-induced callus retains shoot regeneration potency

**Figure 2:**
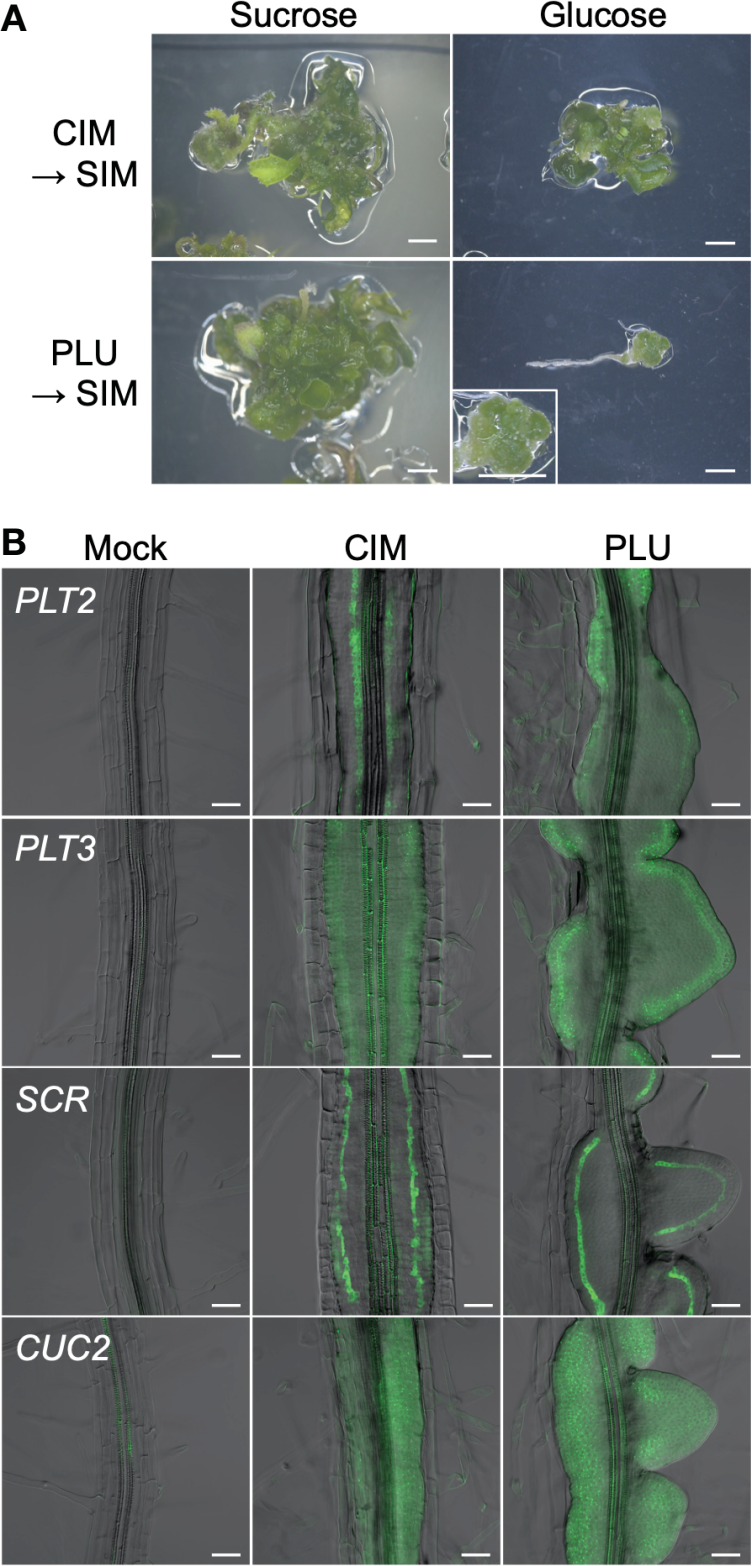
PLU-induced callus retains shoot regeneration potency. **(A)** Root explants from wild-type seedlings at 5 dpi were cultured on CIM or 40 µM PLU-containing medium with sucrose or glucose for 6 days, transferred to SIM, and further cultured for 19 days. The enlarged image in the lower-right panel shows a mass of green cells that did not develop further. Scale bars: 2 mm. **(B)** Roots explants from seedlings of reporter lines at 5 dpi were cultured on PLU-supplemented medium or CIM for 6 days. Fluorescent signals of *PLT2pro:Eluc-3xVenus*, *PLT3pro:Eluc-3xVenus SCRpro:4xYFP*, and *CUC2pro:Eluc-3xVenus* reporters were merged with DIC images. Scale bars: 50 µm.

Expression of *SCR* and *J0121* is known as characteristics of pluripotent callus that retains shoot regeneration potency (Sugimoto et al., 2010; Ikeuchi et al., 2019; Shin et al., 2020). Furthermore, callus formation shares processes with lateral root formation, such as being initiated by the division of XPP cells (Atta et al., 2009; Sugimoto et al., 2010). Because the PLU-induced callus expressed *SCR* and *J0121* (**Figure 1G**) and resembled lateral root primordia (**Figure 1D**), the idea was raised that the PLU-induced callus may retain pluripotency for shoot regeneration like a callus induced by a conventional CIM containing auxin and cytokinin. To test this idea, root explants from wild-type seedlings were cultured for 6 days on CIM or the standard 1/2 MS medium supplemented with 40 µM PLU, and then transferred to cytokinin-rich SIM. After culturing on SIM, green shoot tissues were regenerated in either case (**Figure 2A**, left). This result showed that PLU-treated explants acquired pluripotency to regenerate shoots and that, unlike the conventional CIM, PLU did not require either externally applied auxin or cytokinin for the pluripotency induction. In these experiments, we used sucrose as a sugar source during the CIM or PLU treatments, while glucose can also be used instead of sucrose for CIM (Atta et al., 2009; Sugimoto et al., 2010). We next examined whether the sugar type influences the induction of shoot regeneration potency by PLU (**Figure 2A**). When glucose was used for CIM- or PLU-treatment, CIM-treated explants regenerated shoot tissues on SIM, but not PLU-treated ones. PLU-treated explants only formed a mass of green cells that did not develop further. These results indicated that PLU shows the sucrose preference as a sugar source for the induction of regeneration potency. It is known that CIM-induced callus expresses genes important for establishing pluripotency through lateral root initiation processes, such as *PLT2*, *PLT3*, *SCR*, and *CUP SHAPED COTELYDON 2* (*CUC2*) (Gordon et al., 2007; Kareem et al., 2015; Kim et al., 2018). PLU-induced callus expressed these genes, like CIM-induced callus (**Figure 2B**).

### Rapidly upregulated genes after PLU treatment include a significant number of auxin-response-related genes

To investigate PLU-initiated early events, we performed RNA-seq analysis 1 hour after treatment of wild-type seedlings with PLU and identified 292 genes upregulated more than two-fold (FDR<0.01) (**Dataset S1**). Gene ontology (GO) enrichment analysis showed that the GO categories, “response to endogenous stimulus”, “response to hormone”, and “response to auxin” were enriched with extremely low P-values (**Table S3**). In the hierarchy of GO terms, “response to endogenous stimulus” and “response to hormone” includes “response to auxin” as a subcategory (child category), indicating that “response to auxin” could be the most intrinsically enriched category. When we compared the PLU-upregulated genes with the publicly available list of genes induced by IAA treatment (**Dataset S1**) (Uchida et al., 2018), 82 of 292 PLU-induced genes overlapped with the IAA-induced genes, including well-known auxin-inducible genes, 22 *SMALL AUXIN UP RNA* genes, 8 *IAA/AUX* genes, 4 *GRETCHEN HAGEN 3* (*GH3*) genes, and 3 *LATERAL ORGAN BOUNDARIES-DOMAIN* genes (Okushima et al., 2007; Paponov et al., 2008). These results suggested that the activation of auxin responses could be involved in the action of PLU.

### PLU slowly activates *DR5* responses differently from IAA

**Figure 3:**
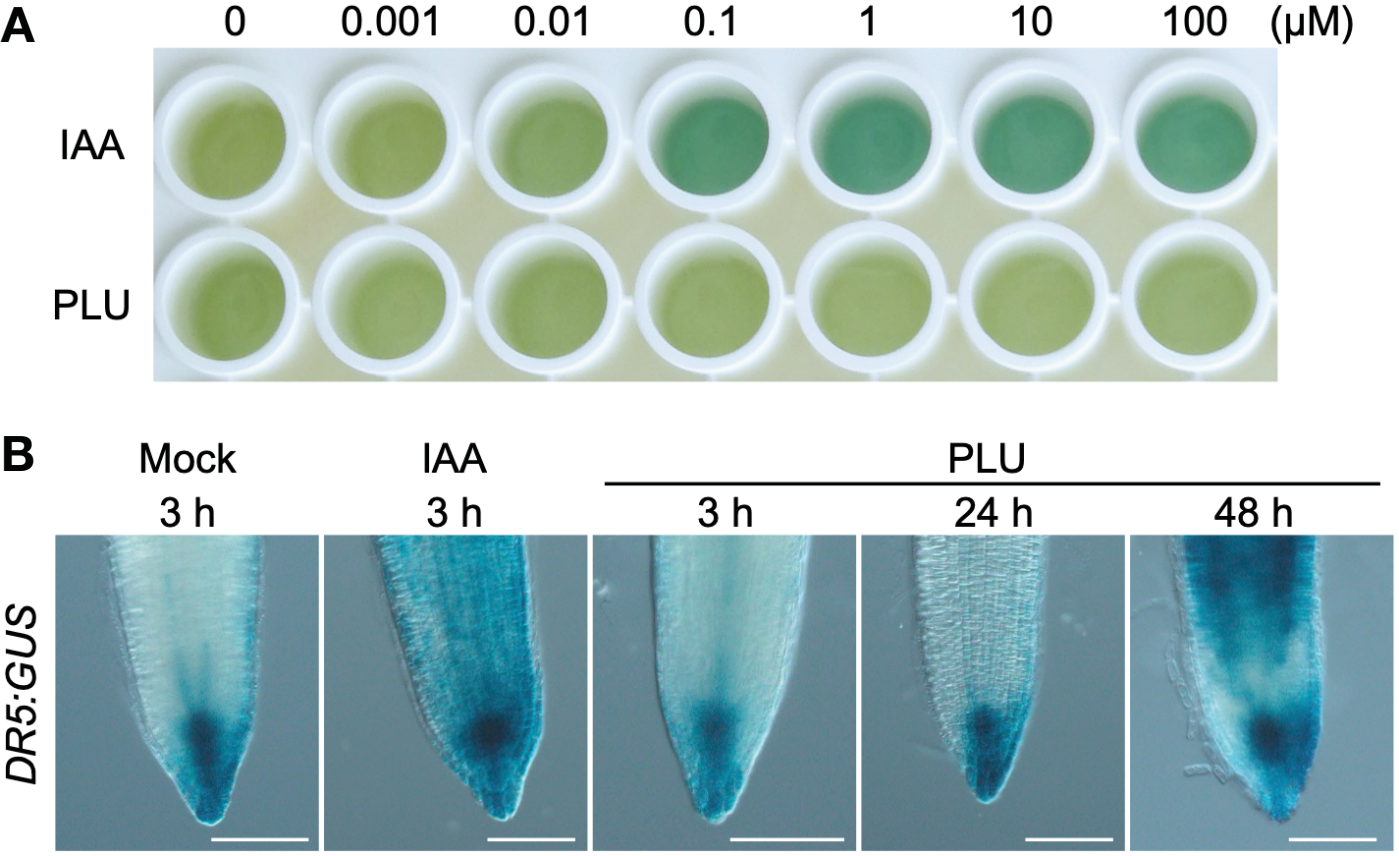
PLU slowly activates *DR5* responses differently from IAA. **(A)** IAA and PLU at various concentrations were applied to the yeast two-hybrid assay that shows the TIR1-AUX/IAA interaction by blue color. **(B)** 5-dpi *DR5:GUS* seedlings were treated with DMSO (mock), 0.5 µM IAA, or 40 µM PLU. GUS staining was performed at indicated time points after compound treatment. Scale bars: 100 μm.

The induction of a significant number of auxin-response-related genes by PLU raised the possibility that PLU may act as an auxin agonist. Auxin regulates downstream responses *via* its receptors, TRANSPORT INHIBITOR RESPONSE 1 (TIR1) and AUXIN SIGNALING F-BOX (AFB) family proteins (Dharmasiri et al., 2005a; Dharmasiri et al., 2005b; Kepinski and Leyser, 2005; Parry et al., 2009; Prigge et al., 2020). Auxin binding to TIR1 promotes the interaction between TIR1 and AUXIN/INDOLE-3-ACETIC ACID (AUX/IAA) proteins (Tan et al., 2007). We examined whether PLU directly binds to TIR1 as an auxin agonist by the previously reported yeast two-hybrid assay that detects the TIR1-AUX/IAA protein interaction (Uchida et al., 2018). Indole-3-acetic acid (IAA), a major natural auxin, effectively promoted the interaction as low as 0.1 µM, while PLU showed no effects even at 100 µM (**Figure 3A**). This result suggested that PLU does not bind to TIR1, consistent with no apparent similarity in molecular structure between PLU and IAA (**Figure 1A**).

Next, we compared the time of the *DR5:GUS* activation at the root tip after IAA or PLU treatments (**Figure 3B**). Strong GUS signals were detected at the entire root tip 3 hours after the treatment of 0.5 µM IAA, showing that, as expected, IAA rapidly activates auxin responses. By contrast, the application of 40 µM PLU, which is sufficient for the induction of callus formation (**Figure 1**), induced no apparent changes in 3 hours and even 24 hours, and GUS signals were eventually enhanced after 48 hours, indicating that PLU slowly activates *DR5*-monitored auxin responses differently from IAA.

### PLU requires an auxin-mediated transcriptional pathway to induce callus formation despite reducing endogenous IAA

**Figure 4:**
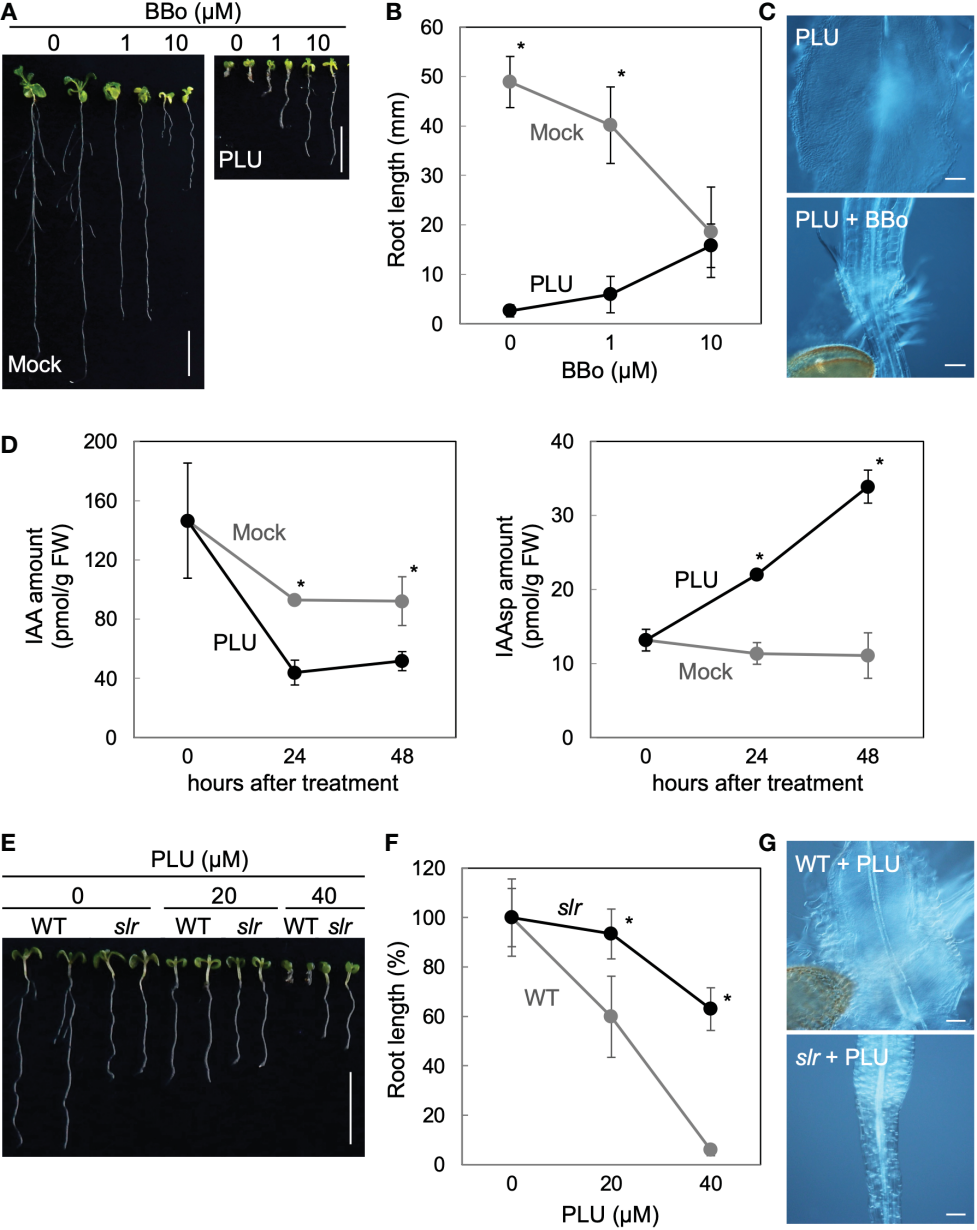
PLU requires an auxin-mediated transcriptional pathway to induce callus formation despite reducing endogenous IAA. **(A–C) (A)** Photos of 8-dpi wild-type seedlings grown on media containing indicated concentrations of BBo with or without 40 µM PLU (Scale bars: 1 cm), **(B)** the quantification graph of root length data in **(A)**, and **(C)** DIC images at the hypocotyl-root junction of 40 µM PLU-treated seedlings cultured with or without 10 µM BBo (Scale bar: 100 µm). **(D)** Quantification graphs of amounts of IAA and its aspartic-acid-conjugated form (IAAsp) per fresh weight (FW) of samples. 5-dpi wild-type seedlings were treated with 40 μM PLU and harvested for quantification at indicated times. **(E–G) (E)** Photos of 8-dpi wild-type or *slr* seedlings grown on media containing indicated concentrations of PLU (Scale bar: 1 cm), **(F)** the quantification graph of root length data in **(E)**, and **(G)** DIC images at the hypocotyl-root junction of 40 µM PLU-treated seedlings (Scale bar: 100 µm). The average root length without compound treatment is set at 100% in **(F)**. In graphs, the mean ± standard deviation (n=10 for **B** and **F**, or n=3 for **D**) is shown. * indicates significant differences between mock and PLU treatments, or WT and *slr* at P < 0.05 based on Welch’s t test (two-tailed).

Because the slow activation of *DR5* responses by PLU could be mediated through the action of endogenous auxin, we investigated how the inhibition of endogenous auxin biosynthesis affects PLU-induced phenomena. When auxin biosynthesis was attenuated by the treatment of 4-biphenylboronic acid (BBo), an inhibitor of YUCCA-family auxin biosynthesis enzymes (Kakei et al., 2015), BBo alleviated root shortening by PLU in a dose-dependent manner and also blocked callus formation at 10 µM (**Figures 4A-C**). Another inhibitor of YUCCA enzymes, Yucasin (Nishimura et al., 2014), which is structurally unrelated to BBo, also suppressed the PLU-induced root shortening (**Figure S2**). These results showed that endogenous auxin is required for PLU-induced effects. Next, we quantified amounts of IAA and its inactivated form in PLU-treated plants. IAA, a major active form of auxin, is known to be converted into inactive aspartic-acid-conjugated form (indole-3-acetyl-aspartate: IAAsp) by GH3-family enzymes (Staswick et al., 2005; Kramer and Ackelsberg, 2015). PLU treatment decreased the IAA amount within 24 hours and increased the IAAsp amount (**Figure 4D**), consistent with our RNA-seq analysis showing that PLU treatment upregulates the expression of *GH3* genes. Since PLU requires endogenous auxin to exert its effects despite the reduction of endogenous IAA, it is likely that PLU enhances sensitivity to auxin.

PLU ectopically activated *DR5* (**Figure 3**), which is a transcriptional output reporter of auxin signaling (Ulmasov et al., 1997). To examine whether PLU requires the auxin-mediated transcriptional pathway to induce callus formation, we applied PLU to the *solitary root/indole-3-acetic-acid 14* (*slr/iaa14*) mutant, in which the auxin-triggered transcriptional activation is attenuated due to a dominant mutation in *IAA14* (Fukaki et al., 2005). The *slr* mutation markedly suppressed both root shortening and callus formation induced by 40 µM PLU (**Figures 4E-G**), showing that auxin-regulated transcription mediates PLU effects.

### PLU requires HSP90 activity to trigger *DR5* responses and callus formation

**Figure 5:**
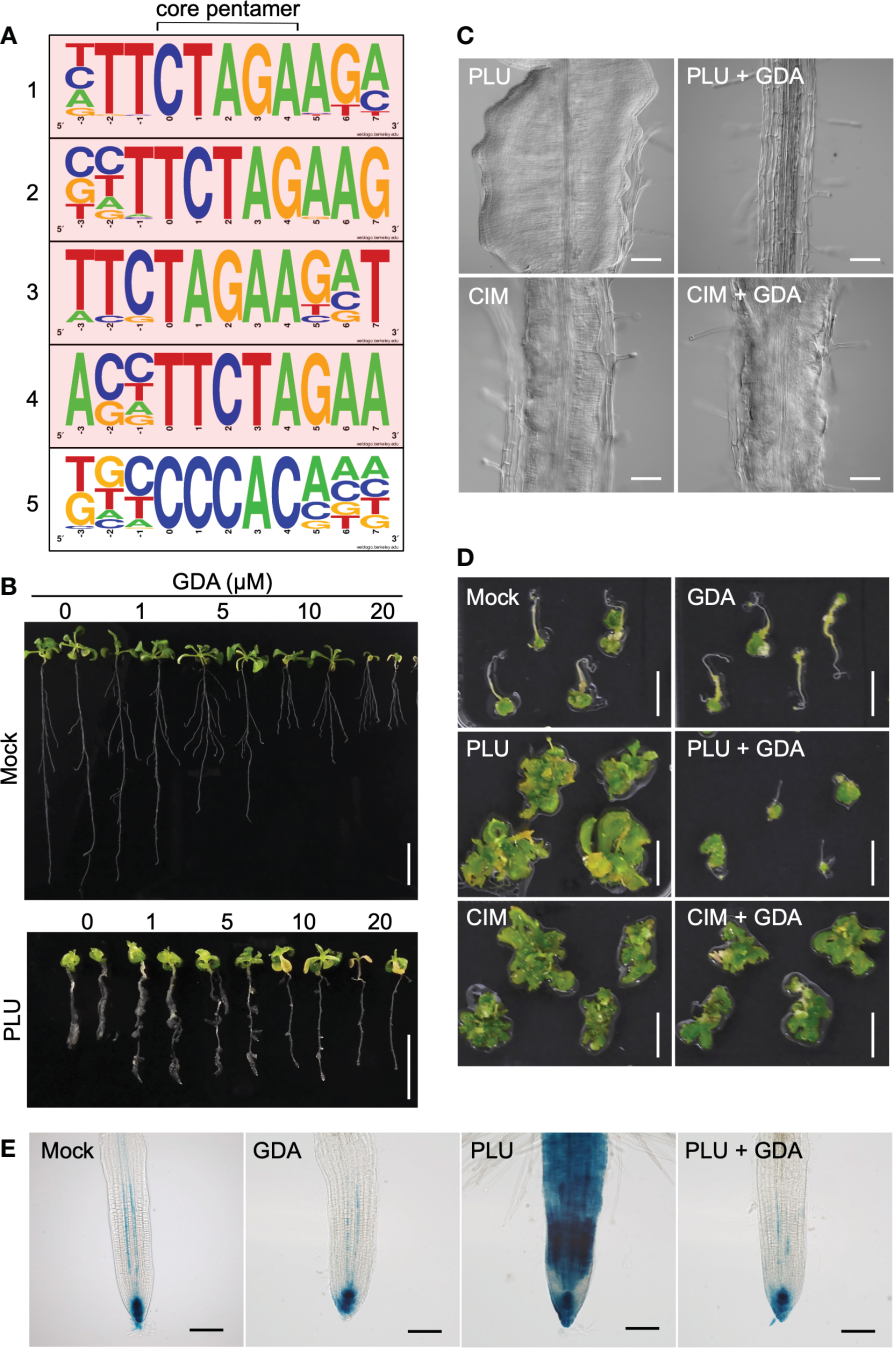
PLU requires HSP90 activity to trigger *DR5* responses and the callus formation. **(A)** The top five motifs overrepresented in 1-kb upstream sequences of 292 genes upregulated more than two-fold 1 hour after PLU treatment by a program that identifies overrepresented cis elements by comparing surrounding sequences for every pentamer in input sequences. The core pentamer of each overrepresented motif is positioned at the center. The top four motifs containing TTCTAGAA sequences are highlighted in pink. **(B)** 5-dpi wild-type seedlings were transferred to media containing indicated concentrations of GDA with or without 40 µM PLU, and observed at 14 dpi. Scale bars: 1 cm. **(C)** DIC images of roots of 12-dpi seedlings. 5-dpi wild-type seedlings grown in normal media were transferred to media containing 40 µM PLU- or CIM with or without 20 µM GDA. Scale bars: 100 µm. **(D)** Root explants from wild-type seedlings at 5 dpi were cultured on 40 µM PLU- or CIM-containing medium with or without 20 µM GDA for 6 days, transferred to SIM, and observed 22 days after the transfer to SIM. Scale bar: 1 cm. **(E)** 5-dpi *DR5:GUS* seedlings were treated with or without 40 µM PLU and/or 20 µM GDA, and GUS staining was performed after 48-hour incubation. Scale bar: 40 µm.

The rapid auxin-related responses within 1 hour and the slow *DR5*-monitored auxin responses may be mediated through distinct regulation. When we drew the Venn diagram showing the number of genes overlapping among the PLU-induced genes, the IAA-induced genes, and also publicly available lists of CIM-induced genes (Sugimoto et al., 2010) and lateral-root-initiation (LRI)-related genes (Vanneste et al., 2005) (**Figure S3, Dataset S1**), the majority of the PLU-induced genes (184 of 292 genes) did not overlap with genes in the compared lists, suggesting that PLU may activate some specific mechanisms to control these downstream genes. To further explore a PLU-initiated mechanism, we next surveyed cis-regulatory elements overrepresented in upstream promoter regions of the PLU-upregulated genes (Yamamoto et al., 2011). Strikingly, 52 genes shared the most overrepresented sequence TTCTAGAA, which preferred a G at its 3’ position (**Figures 5A**, **S4**, **Dataset S1**). By contrast, only 1 of the 81 PLU-downregulated genes retained the TTCTAGAA sequence in its 1-kb upstream sequence (**Figure S4**, **Dataset S1**). The TTCTAGAA sequence is known as Heat Shock Element (HSE), a recognition motif of Heat Shock Factors (HSFs) (Guhathakurta et al., 2002; Manuel et al., 2002). Because Heat Shock Protein 90 (HSP90) plays a central role in regulating HSFs (Scharf et al., 2012; Guo et al., 2016; Jacob et al., 2017), the overrepresentation of HSE in upstream sequences of PLU-induced genes suggested the involvement of HSP90 in the initial action of PLU.

To examine whether PLU requires HSP90 activity to trigger downstream events, we investigated how the reduction of HSP90 activity influences PLU-induced phenomena using Geldanamycin (GDA), a specific inhibitor of HSP90 proteins without known off-target activity (Pearl and Prodromou, 2006; Wang et al., 2016), which has been widely used to overcome the difficulties in the genetic loss-of-function analysis due to high redundancy of *HSP90*-family genes (Stebbins et al., 1997; Krishna and Gloor, 2001; Queitsch et al., 2002; Wang et al., 2016). When wild-type plants were treated with GDA from 1 to 14 dpi, no obvious growth inhibition was observed even with 20 µM GDA (**Figure S5**), indicating that GDA does not retain general non-specific deleterious effects. When 5-dpi wild-type seedlings were transferred to GDA-containing media (**Figure 5B**), the growth of the primary root was attenuated, while lateral roots appeared to be less affected. It is likely that responses to GDA, which may include acclimation or desensitization responses, vary by growth stage and developmental context. The primary root of PLU-treated seedlings grew longer in the presence of GDA than in no GDA condition. GDA treatment completely suppressed PLU-induced callus formation, while CIM induced callus regardless of the presence or absence of GDA (**Figure 5C**). Shoot regeneration experiments, in which PLU- and/or GDA-treated explants were transferred to SIM, showed that PLU did not induce shoot regeneration potency in the presence of GDA (**Figure 5D**). By contrast, CIM-treated samples developed shoot tissues regardless of the presence or absence of GDA (**Figure 5D**), consistent with the result that CIM induced callus even in the presence of GDA. These results indicated that HSP90 activity is required for PLU to induce pluripotency.

It was reported that ectopic activation of auxin signaling by exogenously applied IAA is suppressed by GDA (Wang et al., 2016), raising the possibility that PLU-induced slow activation of auxin responses (**Figure 3**) may also require HSP90 activity. To examine this possibility, *DR5:GUS* seedlings were analyzed 48 hours after PLU treatment in the presence or absence of GDA (**Figure 5E**). In mock-treated seedlings, GUS signals were detected strongly at the root tip and weakly in protoxylem cell files within the stele (De Smet et al., 2007), and GDA did not alter the original *DR5:GUS* pattern. On the other hand, GDA blocked the activation of *DR5:GUS* by PLU, bringing the PLU-induced GUS pattern back to the normal pattern as if there had been no PLU treatment, showing that, although normal auxin responses do not require HSP90 activity, PLU requires it to activate ectopic auxin responses.

### HSP90-dependent induction of auxin receptors is required for callus formation by PLU

**Figure 6:**
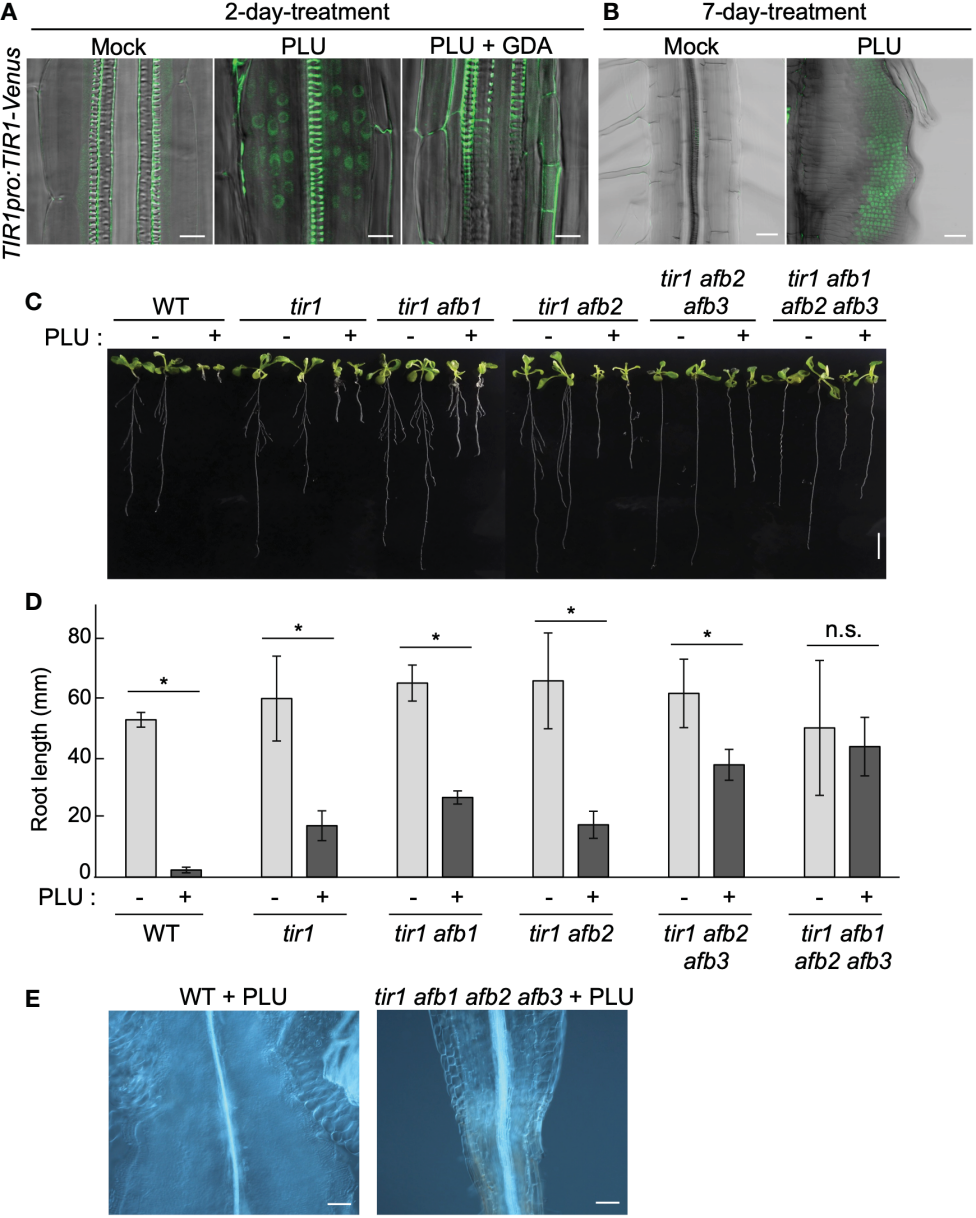
HSP90-dependent induction of auxin receptors is required for callus formation by PLU. **(A, B)** 5-dpi *TIR1pro:TIR1-Venus* seedlings were treated with or without 40 µM PLU and/or 20 µM GDA and observed after 2-day incubation (Scale bar: 50 µm) **(A)** or 7-day incubation (Scale bar: 10 µm) **(B)**. Fluorescent signals were merged with DIC images. Note that xylem strands show strong self-fluorescence due to thick secondary cell wall. **(C)** Photos of 14-dpi wild-type or *tir1/afb*-family mutant seedlings grown on media containing DMSO (mock) or 40 µM PLU (Scale bar: 1 cm). Because some *tir1/afb*-family multiply mutant lines frequently fail to produce the primary root (Dharmasiri et al., 2005b), only seedlings that produced primary root were used for experiments. **(D)** The quantification graph of root length data in **(C)**. The mean ± standard deviation is shown. Sample size is n=8 except for *tir1 afb1 afb2 afb3* (n=6). * indicates significant differences from mock treatment at P < 0.05, and n.s. indicates no significant difference based on Welch’s *t* test (two-tailed). **(E)** DIC images at the hypocotyl-root junction of 40 µM PLU-treated seedlings (Scale bar: 100 µm).

To investigate how HSP90 enables PLU to induce auxin responses, we focused on past reports that a variety of stresses such as heat, osmotic stress, drought, and pathogens affects the expression of *TIR1*/*AFB*-family auxin receptors (Navarro et al., 2006; Chen et al., 2011; Wang et al., 2016; Kalve et al., 2020). It was also reported that HSP90 mediates heat-induced accumulation of TIR1/AFB-family proteins (Wang et al., 2016) and that overexpression of *TIR1* enhances sensitivity to auxin (Chen et al., 2011; Windels et al., 2014). We hypothesized that PLU may induce the expression of *TIR1*/*AFB* family and that this process may require HSP90 activity. To test this hypothesis, we analyzed *TIR1pro:TIR1-Venus* reporter plants. TIR1 proteins localize in the nucleus for transcriptional regulation (Dharmasiri et al., 2005b). PLU treatment of *TIR1pro:TIR1-Venus* seedlings induced nuclear accumulation of Venus signals in cells around vasculatures of roots within 48 hours (**Figure 6A**), in sharp contrast to the mock-treated case that only exhibited strong autofluorescence of thick secondary cell walls of xylem. The nuclear accumulation of TIR1-Venus signals was maintained in several layers of cells of developing callus (**Figure 6B**). GDA blocked the PLU-induced *TIR1-Venus* expression (**Figure 6A**), indicating that PLU requires HSP90 activity to activate *TIR1*. qRT-PCR analysis showed that the 48-hour PLU treatment increased expression levels of endogenous *TIR1* and *AFB* genes (**Figure S6**) and that the co-treatment of GDA suppressed the increase, except for the case of *AFB2*. The expression of *TIR1* and *AFB3* was kept at the level of the mock condition even in the presence of GDA, which is consistent with the result that, although GDA blocked the PLU-induced ectopic activation of auxin responses, it did not cause apparent changes in normal auxin responses (**Figure 5E**). The large reduction of *AFB1* expression by GDA and the resistance of the PLU-induced *AFB2* increase to GDA (**Figure S6**) suggest that the involvement of HSP90 in transcriptional control is not uniform among *TIR1*/*AFB* genes.

To examine the importance of *TIR1/AFB* family for callus induction by PLU, we analyzed how *tir1/afb*-family mutants react to PLU. Because *TIR1/AFB*-family members retain redundant functions (Dharmasiri et al., 2005b; Parry et al., 2009; Prigge et al., 2020), we treated several *tir1/afb*-family multiply mutants with PLU. Higher-order mutants exhibited higher resistance to the PLU treatment, and PLU neither attenuated root growth nor induced callus formation in *tir1 afb1 afb2 afb3* quadruple mutant (**Figures 6C-E**). These results showed that PLU requires *TIR1/AFB* family to induce the formation of pluripotent callus. Collectively, we concluded that PLU potentiates responsiveness to auxin *via* activation of expression of auxin receptor genes.

## Discussion

CIM, which has been used for artificial callus induction, requires externally supplemented auxin and cytokinin to induce pluripotency (Skoog and Miller, 1957; Ikeuchi et al., 2013; Shin and Seo, 2018). Although PLU does not require the external application of either auxin or cytokinin to initiate the formation of pluripotent callus, PLU potentiates responsiveness to endogenous auxin by inducing the expression of auxin receptors. However, only activation of auxin signaling is considered insufficient to induce pluripotency (Skoog and Miller, 1957; Ikeuchi et al., 2013; Shin and Seo, 2018). It was reported that, although *TIR1* overexpression enhances the sensitivity to auxin (Chen et al., 2011; Windels et al., 2014), overexpression of *TIR1* or *AFB*s do not cause callus formation (Chen et al., 2011; Ren et al., 2011; Chen et al., 2015; El-Sharkawy et al., 2016; He et al., 2018; Garrido-Vargas et al., 2020). It is likely that, though auxin responses are required for callus induction, the *TIR1* overexpression alone is insufficient to cause callus induction. Like cytokinin in the case of CIM, another additional factor(s) would be required to effectively induce callus formation. In this regard, the sugar-type preference of PLU for the pluripotency induction may be noteworthy. It is known that the glucose/sucrose ratio affects growth and development and that sugar signaling is complexly intertwined with cytokinin signaling (Wang et al., 2021; Meng et al., 2022). Exploring a mechanism underlying the sugar-type preference of PLU may lead to understanding why PLU can effectively induce pluripotency without the external application of cytokinin.

Cis-element analysis and following experiments revealed the overrepresentation of the HSE motif among PLU-upregulated genes and the requirement of HSP90 for PLU-initiated callus formation. A variety of internal and external stresses, as well as heat stress, modulate HSP90 activity (Sangster and Queitsch, 2005; Kadota and Shirasu, 2012; Di Donato and Geisler, 2019). HSP90 proteins, which pre-exist in cells, initiate downstream events in response to stresses. Because our RNA-seq data indicates no rapid change in *HSP90* expression after PLU treatment, PLU likely modulates the activity of pre-existing HSP90 proteins. The unidentified direct target of PLU may be a factor acting in the machinery that modulates HSP90 activity in response to specific stress. Because HSP90 functions by forming context-specific protein complexes (Sangster and Queitsch, 2005; Kadota and Shirasu, 2012; Di Donato and Geisler, 2019; Toribio et al., 2020), it would be interesting to investigate whether PLU influences HSP90-including protein complexes and what context-specific complex PLU modulates.

PLU induces expression of *TIR1/AFB*-family genes in an HSP90-dependent manner. It was reported that heat stress enhances the TIR1 activity by stabilizing TIR1 proteins *via* HSP90 (Wang et al., 2016). However, despite the overrepresentation of the HSE motif among PLU-upregulated genes, PLU treatment did not induce apparent heat responses such as enhanced elongation of hypocotyls. Because a variety of stresses such as osmotic stress, drought, and pathogens can affect the transcription of *TIR1/AFB*-family genes (Navarro et al., 2006; Chen et al., 2011; Kalve et al., 2020), PLU may modulate some stress-related pathway other than heat. Combined with the report that HSP90 stabilizes TIR1 proteins upon heat stress (Wang et al., 2016), PLU may synergistically activate TIR1 in an HSP90-dependent manner by both the transcriptional level and the protein stability level.

Other possible mechanisms for the mode of action of PLU may include the modulation of auxin transport. True leaves of PLU-treated seedlings exhibited a venation pattern similar to those with defects in auxin transport (Thomson et al., 1973; Sieburth, 1999). Unlike IAA that can directly and globally affect downstream genes and, therefore, can rapidly activate *DR5* responses, PLU may modulate a mechanism that can affect the expression of only a part of auxin-regulated genes (**Figure S3**). Auxin transport could be involved in this partial effect of PLU on auxin-regulated genes.

Although artificial induction of callus by CIM containing auxin and cytokinin is a general procedure for plant transformation and some agricultural applications, the production of pluripotent callus is still challenging for many plant species. Another small compound, fipexide (FPX), was also reported to promote callus formation and shoot regeneration in plants (Nakano et al., 2018), and the mode of action of FPX seems different from that of PLU or CIM because FPX forms pluripotent tissues without vasculatures, while PLU and CIM induce those with vasculatures (**Figure 1F**) (Nakano et al., 2018). Further exploring details of pluripotency-inducing mechanisms activated by these novel compounds, including analysis of which parts or functional groups in their chemical structures are critical for their activities, may lead to developing alternative pluripotency induction methodologies that complement the conventional methodology. Because these compounds contain an amide linkage, which could be cleaved in plants (Savaldi-Goldstein et al., 2008), they may become active forms by being cleaved in plants. It would be noteworthy that their possible chemical structures after the presumable cleavage of the amide linkage can commonly retain the same moiety as the side chain of 2,4-D, a synthetic auxin. In this scenario, the identification of actual *in planta* structures of such auxin-like forms after the cleavage may provide further insights into the understanding of the mode of action of these compounds and facilitate the applied usage of these compounds.

## Data availability statement

The datasets presented in this study are deposited in the DDBJ repository, accession number DRA013685.

## Author contributions

NU conceived the project; YN and NU designed experiments; YN, YK, HE, AH, RI, MK, YT, HS, SK, MN, YT, and NU performed research and analyzed data; MM, RK, RI, AS, KI, SH, KT, and NU developed and provided materials; YN and NU wrote the manuscript; YN, HS, SK, SH, KT and NU edited the manuscript. All authors contributed to the article and approved the submitted version.
