# Supplementary material for: Identification of a pluripotency-inducing small compound, PLU, that induces callus formation via Heat Shock Protein 90-mediated activation of auxin signaling
Source: Front Plant Sci. 2023 Mar 8;14:1099587. doi: 10.3389/fpls.2023.1099587 (PMC10030974; doi:10.3389/fpls.2023.1099587)
Supplement: Supplementary file 2 [file DataSheet_2.pdf]

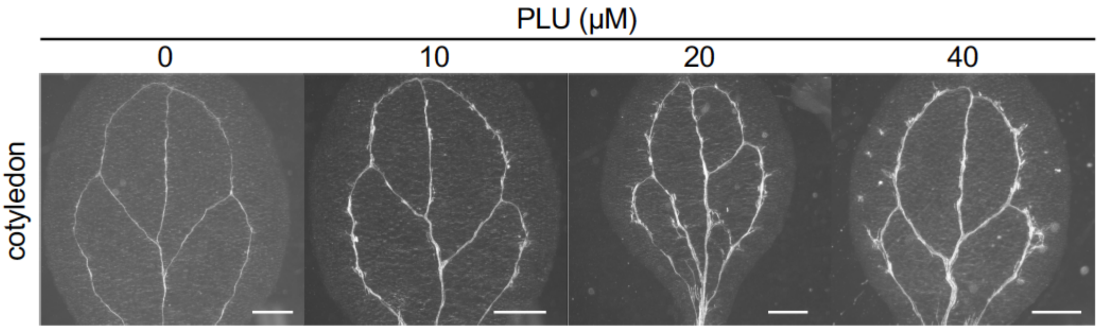

Supplementary Figure S1

Venation patterns of cotyledons of PLU-treated seedlings.

DIC images of cleared cotyledons of wild-type seedlings treated with PLU for 11 days. Scale bars: 200 μm.

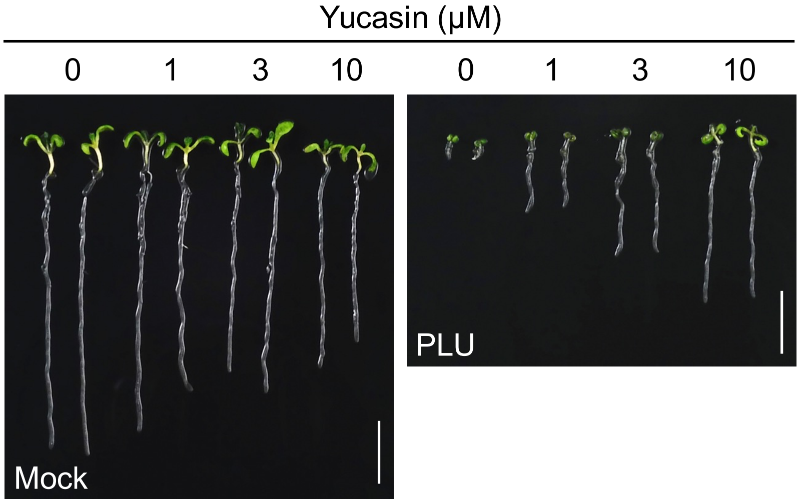

Supplementary Figure S2

Effects of Yucasin on PLU-treated seedlings

Photos of 8-dpi wild-type seedlings grown on media containing indicated concentrations of Yucasin with or without 40 µM PLU from 1 to 8 dpi (Scale bars: 1 cm).

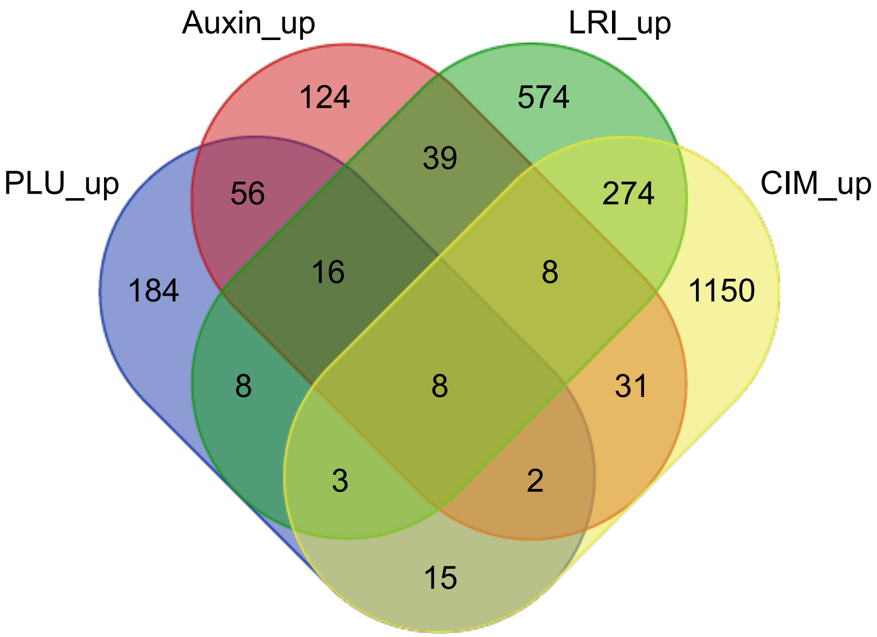

Supplementary Figure S3

Venn diagram of PLU-upregulated genes and the previously reported gene lists.

Venn diagram was drawn using PLU-upregulated genes (PLU_up) by our RNA-seq analysis and the publicly available lists of auxin-induced genes (Auxin_up), CIM-induced genes (CIM_up), and Lateral Root Initiation-related genes (LRI_up) (Sugimoto et al., 2010; Uchida et al., 2018; Vanneste et al., 2005).

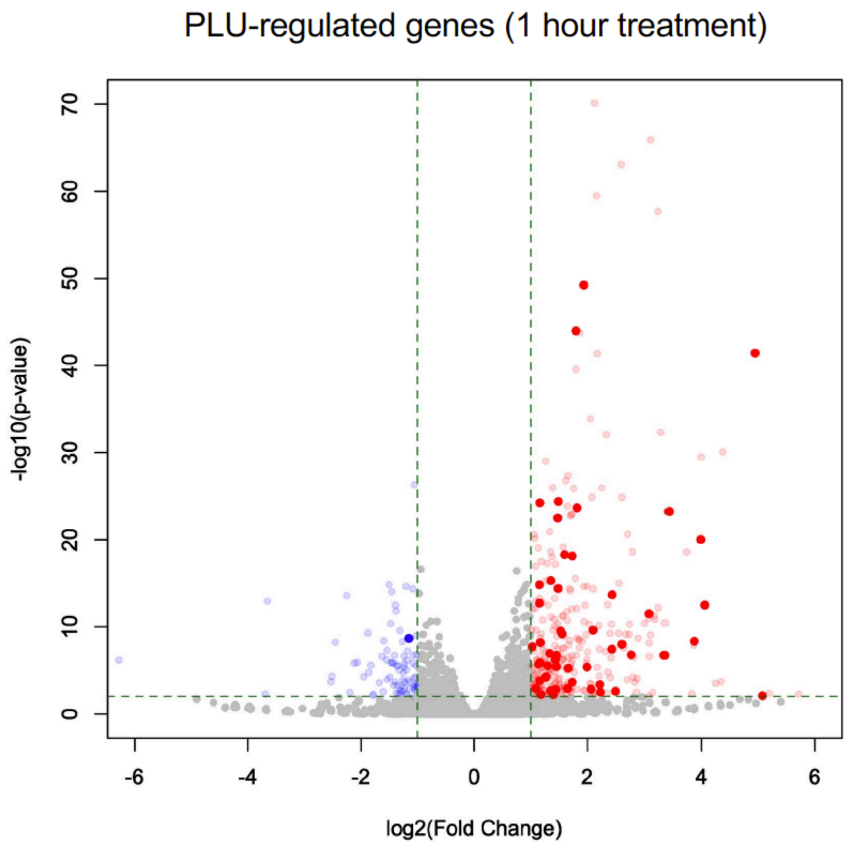

Supplementary Figure S4

Vorcano blot of up- and down-regulated genes by 1 hour PLU treatment

The fold change (log2) is plotted on the x-axis, and FDR (p-value, the negative log10) is plotted on the y-axis by comparing RNA-seq data of 1 hour PLU treatment with mock treatment. Colored dots indicate genes with more than 2-fold expression change (FDR<0.01). Up- and down-regulated genes are shown in blue and red. Respectively. Dots with dark color indicate HSE-containing genes.

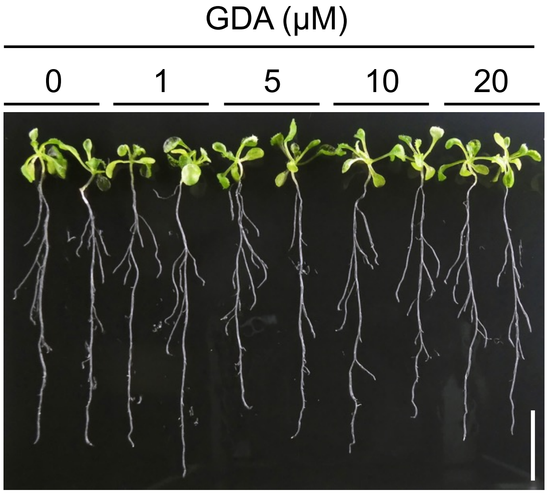

Supplementary Figure S5

Effects of GDA on seedling griowth

Wild-type seedlings were grown with indicated concentrations of GDA from 1 to 14 dpi and observed at 14 dpi. Scale bars: 1 cm.

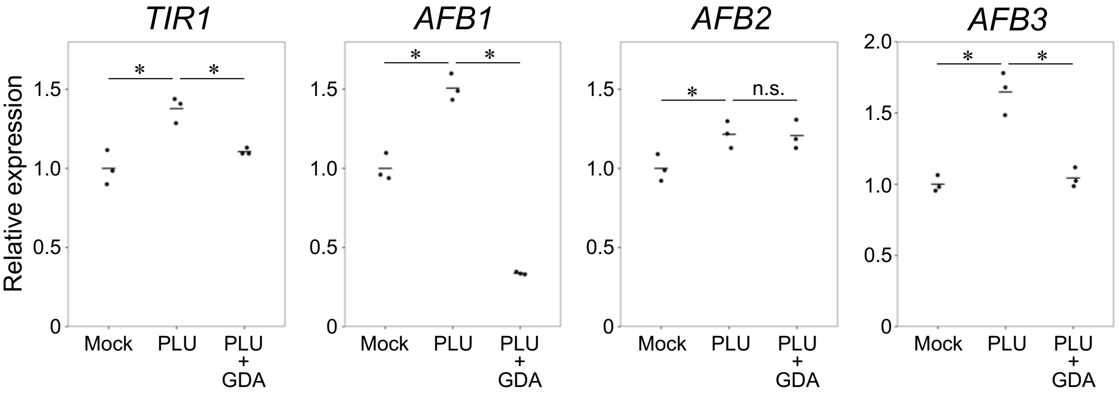

Supplementary Figure S6

Expression levels of *TIR1*/*AFB* genes after PLU treatment

Relative expression levels of *TIR1*/*AFB* genes normalized against *ACTIN8* expression were determined by qRT-PCR. 5-dpi wild-type seedlings were treated with or without 40 µM PLU and/or 20 µM GDA for 2 days, and total RNA from whole seedlings was subjected to qRT-PCR. Dots and crossbars indicate data points of individual samples and the mean of three biological replicates, respectively, in each condition. The mean value of mock-treated samples is set at 1. Asterisks and n.s. indicate significant differences at $P < 0.05$ and no significant difference, respectively, based on Welch's *t*-test (two-tailed).

Supplementary Table S1

List of plasmids constructed in this study

Plasmid	Bacteria	Plant	Note
NOSt in *pAN19*	Amp	-	*NOS* terminator was amplified from pBI101 by PCR
linker-3xVenus:NOSt in *pAN19*	Amp	-	*linker-3xVenus* was transferred from *pDONR/Zeo:cENF2-3xVenus*
linker-3xVenus:NOSt in *pBIN50*	Kan	Kan	*linker-3xVenus:NOSt* was transferred from *pAN19/linker-3xVenus:NOSt*
ELuc-3xVenus:NOSt in *pBIN50*	Kan	Kan	*ELuc* CDS was amplified from *pEluc-PEST* by PCR
PLT2pro:ELuc-3xVenus:NOSt in *pBIN50*	Kan	Kan	*PLT2* promoter was amplified from Col genome by PCR
PLT3pro:ELuc-3xVenus:NOSt in *pBIN50*	Kan	Kan	*PLT3* promoter was amplified from Col genome by PCR
CUC2pro:ELuc-3xVenus:NOSt in *pBIN50*	Kan	Kan	*CUC2* promoter was amplified from Col genome by PCR
linker-Venus:NOSt in *pAN19*	Amp	-	*linker-Venus* was amplified from *pBIN50/linker-3xVenus:NOSt* plasmid by PCR
TIR1pro:TIR1-Venus:NOSt in *pAN19*	Amp	-	*TIR1* promoter and *TIR1 cDNA* was amplified from Col genome and Col total cDNA by PCR
TIR1pro:TIR1-Venus:NOSt in *pBIN30*	Kan	BASTA	*TIR1pro:TIR1-Venus:NOSt* was transferred from *pAN19/TIR1pro:TIR1-Venus:NOSt*

Supplementary Table S2

List of primers used in this study

Amplicon	Primer name	Sequence
NOS terminator	NOS-ter-SacI-F	TACCGAGCTCTCCCCGATCGTTCAAACATTTG
	NOS-ter-R	TAGAATTCGATCTAGTAACATAGATGACACC
ELuc CDS	ELuc-F-NEB	AAGCTTCCCGGGGTCGACTGGATCCATGGAGAGAGAGAAGAACGTGGTGTAC
	ELuc-R-linker-NEB	TCACACCACCGGTCGAGGGGCTGCCTCCGCCGCTGCCTCCGCCCAGCTTAGAAGCCTTCTCCATCAG
PLT2 promoter	PLT2-pro-F-NEB	CTATGACCATGATTACGCCATATCAGGTTTAGAGAATTCTCAAGAAATAGTC
	PLT2-pro-R-NEB	ACGTTCTTCTCTCTCTCCATTTCCCTTTTCTTGGAATCAAAGCTTAAAC
PLT3 promoter	PLT3-pro-F-NEB	CTATGACCATGATTACGCCACTTTCCATGTACTACTACTACATATCAATTGTG
	PLT3-pro-R-NEB	ACGTTCTTCTCTCTCTCCATAAACTTTCTTATAAAAACAATTTTACTTTTTCTCTCTC
CUC2 promoter	CUC2-pro-F-NEB	CTATGACCATGATTACGCCAGGTAGGGATCGAGTCTGAGAAAG
	CUC2-pro-R-NEB	ACGTTCTTCTCTCTCTCCATTAAGAAGAAAGATCTAAAGCTTTTGTTTGAGAG
linker-Venus	linker-Venus-F-XhoI	CAACCTCGAGGGTGGCTCGACCGGTGGTG
	Venus-R-SacI	TACCGAGCTCCTACTTGTACAGCTCGTCCATG
TIR1 promoter	TIR1-pro-F-SalI	CAACGTCGACGAGTACGAAACCCGAGACTAGGAG
	TIR1-pro-R-BamHI	CGGGATCCTGCGGCCAAATAACCTCGAG
TIR1 CDS	TIR1-SmaI-F	TCCCCCGGGCCGCAATGCAGAAGCGAATAGCCTTG
	TIR1-nonstop-SalI-R	CAACGTCGACTAATCCGTTAGTAGTAATGATTTGCCTG
ACT8 qRT-PCR	ACT8-qF	CAAGGAGAAGCTTTCCTTTGTC
	ACT8-qR	AGTTGTAAGTTGTCTCGTGGAT
TIR1 qRT-PCR	TIR1-qF	GCCTTTTGTCATGGAACCAAAT
	TIR1-qR	ATTTGACGGCAGAAGTAGAGAA
AFB1 qRT-PCR	AFB1-qF	CTTATCTGGTCTCTGGGATGTC
	AFB1-qR	TTGTCCTCAATCAAGTCCATCA
AFB2 qRT-PCR	AFB2-qF	GAGCAAGTATGAAACAATGCGA
	AFB2-qR	CAGTCCGGTACAGATACAACTT
AFB3 qRT-PCR	AFB3-qF	AAAACTGTCCAAACTTCATCCG
	AFB3-qR	GCGTACATACCGATGTAGAGAA

Supplementary Table S3

List of enriched GO categories of PLU-upregulated genes

GO category with more than 3-fold enrichment

GO category	Number of genes	Fold enrichment	P-value
response to endogenous stimulus (GO:0009719)	108	4.1	6.23E-35
response to hormone (GO:0009725)	105	4.04	3.69E-33
response to auxin (GO:0009733)	47	11.79	1.45E-30
response to organic substance (GO:0010033)	119	3.28	3.16E-30
cellular response to hypoxia (GO:0071456)	27	10.91	1.35E-15
cellular response to decreased oxygen levels (GO:0036294)	27	10.82	1.64E-15
cellular response to oxygen levels (GO:0071453)	27	10.78	1.80E-15
response to hypoxia (GO:0001666)	30	8.97	2.11E-15
cellular response to chemical stimulus (GO:0070887)	70	3.3	2.46E-15
response to decreased oxygen levels (GO:0036293)	30	8.75	3.96E-15
response to oxygen levels (GO:0070482)	30	8.7	4.62E-15
response to abscisic acid (GO:0009737)	40	3.6	1.66E-08
response to alcohol (GO:0097305)	42	3.4	2.76E-08
regulation of hormone levels (GO:0010817)	21	6.65	9.06E-08
cellular response to endogenous stimulus (GO:0071495)	40	3.24	3.75E-07
response to water deprivation (GO:0009414)	35	3.38	2.02E-06
response to acid chemical (GO:0001101)	37	3.23	2.08E-06
cellular response to hormone stimulus (GO:0032870)	37	3.09	6.95E-06
response to water (GO:0009415)	35	3.15	1.18E-05
response to osmotic stress (GO:0006970)	31	3.35	2.56E-05
response to organonitrogen compound (GO:0010243)	25	3.73	1.09E-04
defense response to bacterium (GO:0042742)	32	3	1.69E-04
regulation of phenylpropanoid metabolic process (GO:2000762)	7	23.31	2.18E-04
response to fungus (GO:0009620)	31	3.01	2.67E-04
response to nitrogen compound (GO:1901698)	26	3.18	1.15E-03
regulation of secondary metabolic process (GO:0043455)	9	10.35	1.52E-03
toxin metabolic process (GO:0009404)	10	8.19	2.61E-03
defense response to fungus (GO:0050832)	23	3.16	5.98E-03
cytokinin catabolic process (GO:0009823)	4	64.39	6.61E-03
response to light intensity (GO:0009642)	16	4.14	9.37E-03
hormone catabolic process (GO:0042447)	4	48.29	1.53E-02
response to chitin (GO:0010200)	14	4.39	2.08E-02
auxin transport (GO:0060918)	8	7.88	4.27E-02
